# Pacinian Corpuscle‐Inspired Strain Conversion Enables Ultrasensitive, Linear, and Broad‐Range Piezoelectric Sensing for Cardiovascular Health Monitoring

**DOI:** 10.1002/advs.202522157

**Published:** 2026-02-03

**Authors:** Qi Yang, Zhongqian Song, Shengjie Liu, Yingming Ma, Weiyan Li, Huijun Kong, Cuiyu Liu, Yu Bao, Li Niu

**Affiliations:** ^1^ Center for Advanced Analytical Science c/o School of Chemistry and Chemical Engineering Guangzhou University Guangzhou P. R. China; ^2^ College of Medical Information and Artificial Intelligence Shandong First Medical University & Shandong Academy of Medical Sciences P. R. China; ^3^ School of Chemical Engineering and Technology Sun Yat‐sen University Zhuhai P. R. China

**Keywords:** bioinspired design, cardiovascular health monitoring, piezoelectric sensors, ultrasensitive and linear responses

## Abstract

High‐fidelity wrist pulse acquisition, essential for the early diagnosis and precise management of cardiovascular diseases, requires tactile sensors with both ultrasensitive and linear electromechanical responses. Biological Pacinian corpuscles transduce mechanical stimuli into localized strain via concentric lamellar architecture, enabling subtle and dynamic perception of pressure fluctuations. Inspired by the working mode of Pacinian corpuscles, this work presents a piezoelectric tactile sensor featuring a multilayer grooved architecture that transduces external pressure into localized in‐plane strain within the piezoelectric layer, effectively enhancing dipole alignment and charge separation. Finite element simulations and experimental results confirm that the grooved architecture contributes to strain concentration, giving rise to an ultrahigh sensitivity of 185 mV·kPa^−^^1^ and linear electromechanical response up to 300 kPa and a power density of 806 µW·cm^−^^2^. The tactile sensor enables high‐fidelity acquisition of multi‐site pulse waveforms and accurate estimation of blood pressure, facilitating comprehensive cardiovascular assessment via heart rate variability and Poincare analysis. This bioinspired design offers an effective approach to overcoming the intrinsic limitations of piezoelectric materials and holds significant potential for developing high‐performance piezoelectric sensors for continuous, noninvasive health monitoring.

## Graphical abstract

A bioinspired piezoelectric sensor mimicking Pacinian corpuscles is developed to enable ultrasensitive and linear pressure sensing. A multilayer grooved architecture converts normal pressure into in‐plane strain, delivering high sensitivity, wide linear range, and efficient energy harvesting, enabling high‐fidelity wrist pulse monitoring and noninvasive cardiovascular health assessment.

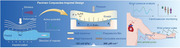

## 1 Introduction

Continuous, noninvasive monitoring of cardiovascular health is essential for the early diagnosis and effective management of cardiovascular diseases. High‐fidelity wrist pulse waveforms acquisition and analysis enable the reasonable assessment of blood pressure, arterial stiffness, ejection capacity, and heart rate variability, providing valuable insights into the physiological status of the cardiovascular system. High‐fidelity acquisition of pulse waveforms requires tactile sensors with both ultrasensitive and linear electromechanical responses, contributing to accurate monitoring of subtle changes in physiological and hemodynamic status. Compared with sensors based on capacitive [1], piezoresistive [2, 3], and triboelectric [4] mechanisms, piezoelectric tactile sensors have attracted significant attention due to their self‐powered operation, rapid response, and mechanical adaptability [5]. However, the output performance of piezoelectric sensors still faces challenges in achieving sufficient sensitivity and wide detection range due to the intrinsically low piezoelectric coefficients and high elastic modulus of piezoelectric materials, which restrict mechanical deformation and suppress charge output, resulting in compromised sensitivity and limited detection range [6].

To overcome these challenges, various strategies have been proposed to optimize the output performance of devices [7, 8]. For instance, integrating electret films can significantly enhance piezoelectric coefficients but often suffers from poor long‐term stability [9]. Incorporating high‐coefficient piezoelectric materials into flexible polymers can improve the piezoelectric response but often reduces their flexibility and mechanical robustness [10]. Meanwhile, conventional piezoelectric sensors based on planar compression structures usually exhibit low sensitivity due to the limited vertical deformation in the piezoelectric film. Cantilever‐beam structures have been proposed to transduce the vertical loading into bending deformation at the free end for strain amplification and output improvement. However, the localized stress concentration could cause plastic deformation or mechanical failure of the piezoelectric films under high loads, making it difficult to maintain high sensitivity and durability across a wide pressure range. Therefore, improving strain amplification capability while preserving mechanical robustness remains a key challenge for achieving both high sensitivity and wide detection range in piezoelectric tactile sensors.

In biological tactile system, Pacinian corpuscles act as special subcutaneous mechanoreceptors capable of perceiving subtle and dynamic forces. Their concentric lamellar architecture converts external pressure into localized strain in mechanosensitive regions, enabling efficient transduction from mechanical stimulus into neural impulses over a broad dynamic range [11, 12]. Inspired by this natural tactile sensation system, we designed a piezoelectric tactile sensor with a multilayer grooved architecture that transforms vertical loading into in‐plane tensile strain within the piezoelectric layer. This configuration provides a structural route to simultaneously improve sensitivity (185 mV·kPa^−^^1^) and maintain linear response across a wide pressure range (300 kPa). Such a bioinspired approach not only addresses the intrinsic limitations of piezoelectric materials but also offers broad potential for applications in continuous cardiovascular monitoring.

## 2 Results and Discussion

### 2.1 Pacinian Corpuscle‐Inspired Piezoelectric (PCP) Sensor Design and Working Mechanism

**FIGURE 1:**
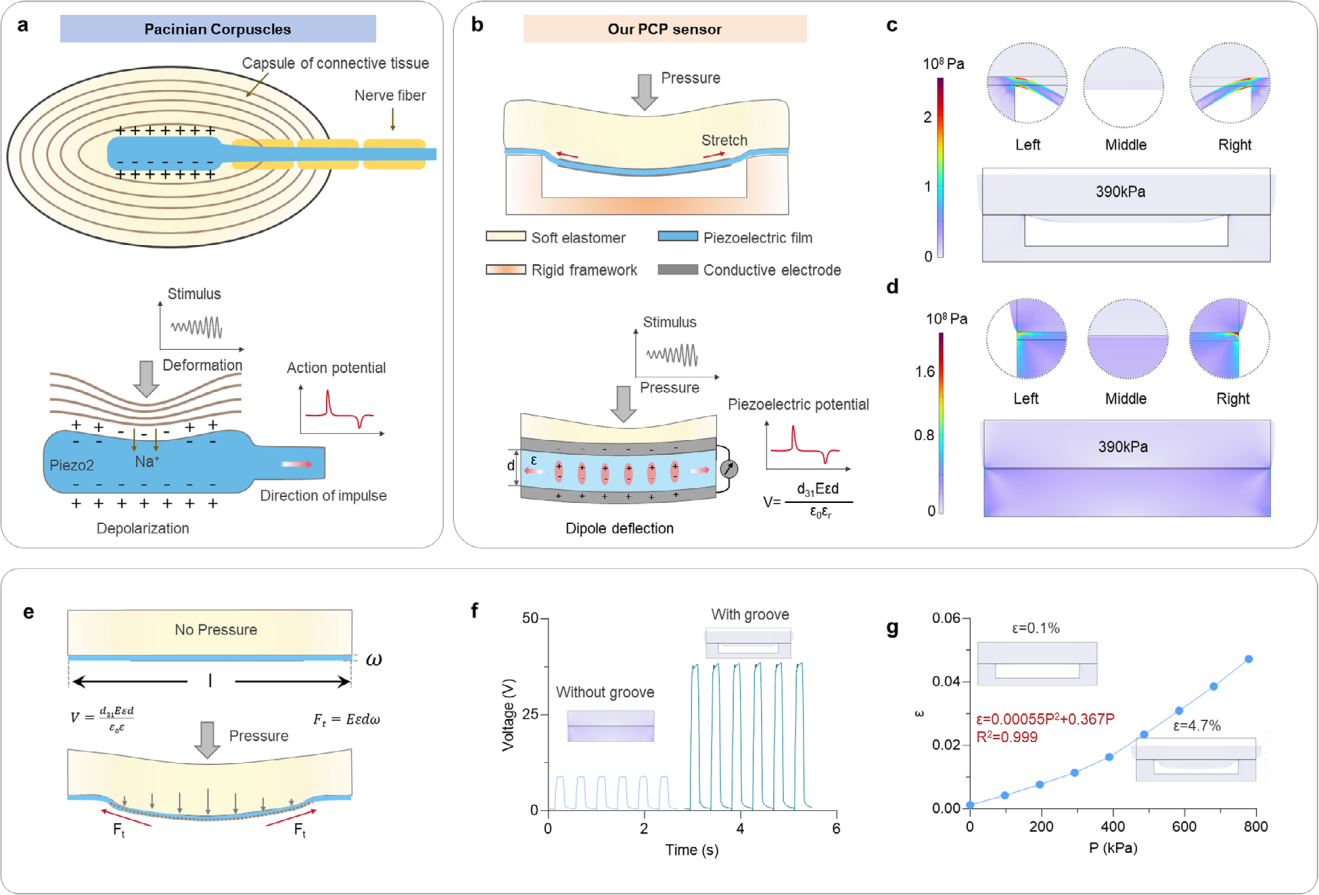
Pacinian corpuscle‐inspired piezoelectric (PCP) sensor design and working mechanism. a) Structural schematic of a biological Pacinian corpuscle, showing nerve fibers encapsulated by concentric lamellar layers of connective tissue. The magnified view depicts the deformation‐induced sodium ion influx and subsequent generation of an action potential, achieving mechanical‐to‐electrical signal conversion. b) Schematic of the PCP sensor comprising a soft elastomer, grooved rigid framework, conductive electrodes, and a suspended piezoelectric film. External pressure deforms the elastomer into the groove, inducing in‐plane stretching of the piezoelectric layer and promoting dipole alignment. Finite element simulations of PVDF piezoelectric sensors c) with and d) without the grooved structure under 390 kPa loading, showing stress distribution at representative positions (left, middle, right). e) Working mechanism and model of the PCP sensor under vertical pressure. f) Comparison of output voltages of piezoelectric sensors with (right) and without (left) grooved structures under 390 kPa, demonstrating the enhanced output voltage enabled by the grooved architecture. g) Relationship between the tensile strain and vertical force.

Pacinian corpuscles are oval‐shaped mechanoreceptors comprising concentric lamellar layers of connective tissue encapsulating a central nerve ending (Figure 1a). The following magnified view illustrates the working mechanism of the Pacinian corpuscle, where mechanical deformation induces depolarization and generates action potentials. Under resting conditions, Pacinian corpuscle neurons maintain a stable membrane potential. Upon mechanical stimulation, deformation of the capsule membrane opens mechanosensitive ion channels, allowing sodium ions influx and the generation of an action potential. This concentric lamellar architecture thus converts external mechanical stimuli into electrical signal.

Inspired by this biological principle, we designed a Pacinian corpuscle‐inspired piezoelectric (PCP) sensor composed of a soft elastomer, a suspended PVDF piezoelectric film, conductive electrodes, and a rigid supporting frame with a rectangular groove (Figure 1b; Figure S1). The soft elastomer provides mechanical buffering and strain transfer, while the groove guides deformation into the suspended piezoelectric PVDF layer. Upon vertical loading, the elastomer compresses into the groove, inducing bending of the PVDF film. This bending generates in‐plane tensile strain of the piezoelectric film, enhancing dipole alignment and charge separation in a d_31_ mode, and thus boosting piezoelectric output (Figure 1b). The piezoelectric output voltage can be described as

(1) $$V=\frac{d_{31}E\varepsilon d}{\varepsilon_{0}\varepsilon_{r}}$$

where d_31_ is the piezoelectric coefficient, *E* is Young's modulus of the piezoelectric film, ε is the tensile strain, d is the film thickness, ε_*r*_ is the relative permittivity, and ε_0_ is the vacuum permittivity. This model establishes a quantitative correlation between deformation and output voltage (Figure S1).

To confirm the advantages of the designed grooved structure, we fabricated a piezoelectric sensor without a grooved design (Figure S2). Finite element simulation (FEA) results revealed that the grooved structure concentrates stress at the film's edges (Figure 1c), achieving a peak stress of 2 × 10^8^ Pa, significantly higher than that observed in the planar configuration (0.5 × 10^8^ Pa, Figure 1d). Stress at different positions (left, center, right) confirmed that the grooved design redistributed the vertical compression into lateral tensile strain, whereas the planar design predominantly experiences vertical compression with limited tangential stress.

Figure 1e illustrates the deformation and working mechanism of the grooved‐structure piezoelectric sensor. Under pressure loading, the elastomer layer deforms into the groove and drives the PVDF film into a bending and stretching mode. The in‐plane stretching‐induced lateral tensile forces (F_t_) promotes more effective dipole reorientation and charge separation, thereby amplifying the voltage output. This grooved design contributes to edge traction and geometric deformation to achieve strain amplification. As for the planar‐structured sensors, the piezoelectric film undergoes compressive deformation only in the vertical direction under normal force, resulting in dipole polarization along the thickness direction and subsequent charge accumulation between the electrodes. However, due to the deformation being confined to the vertical axis, the structure exhibits limited response to subtle tangential stress and therefore contributes to relatively low sensitivity. The corresponding working mode is shown in Figure S3. As illustrated in Figure 1f, the grooved sensor produces an output voltage of 38 V at 330 kPa, four times higher than that of the planar sensor (8.5 V). These results indicate that the grooved structure not only concentrates mechanical stress within the piezoelectric layer but also converts vertical pressure into lateral strain, substantially enhancing the electromechanical response.

Further simulations across a pressure range of 0 to 800 kPa revealed a monotonic increase in tensile strain (Figure 1g). The strain was calculated using the equation

(2) $$\varepsilon =\frac{L-L_{0}}{L_{0}}$$

where *L* and *L*_0_ denote the deformed and original lengths of the piezoelectric film, respectively. The relationship between the tensile strain and vertical force is fitted as ε = 4.2*10^−8^*P^2^ + 0.0000278P. According to Equation (1), the relationship between the output voltage and external pressure can be derived as

(3) $$V=\frac{d_{31}\mathrm{Ed}\left(4.2*10^{-8}P^{2}+2.78*10^{-6}P\right)}{\varepsilon_{0}\varepsilon_{r}}$$

Based on the derived analytical model, the output voltage of the grooved‐architecture piezoelectric sensor can be expressed as V = 0.367P + 0.00055P^2^, where P represents the external pressure (kPa), and V is the output voltage (V). The derivation was performed using typical PVDF parameters (d_31_ = 20 pC/N, E = 2.5 GPa, d = 28 µm, ε_r_ = 12, ε_0_ = 8.854 pF/m). The simplified expression reveals a dominant linear term accompanied by a minor quadratic correction, indicating that the sensor exhibits a nearly linear electromechanical response within the appropriate pressure range, while the quadratic term accounts for higher‐order strain effects under large loads. The strain at high pressure is confined by the groove depth, which not only ensures the linear response but also prevents mechanical damage to the piezoelectric film. This bioinspired approach offers a versatile route for boosting piezoelectric transduction efficiency and holds broad potential for applications in wearable electronics, biomedical diagnostics, and intelligent robotics.

### 2.2 Structural Optimization of the PCP Sensor

**FIGURE 2:**
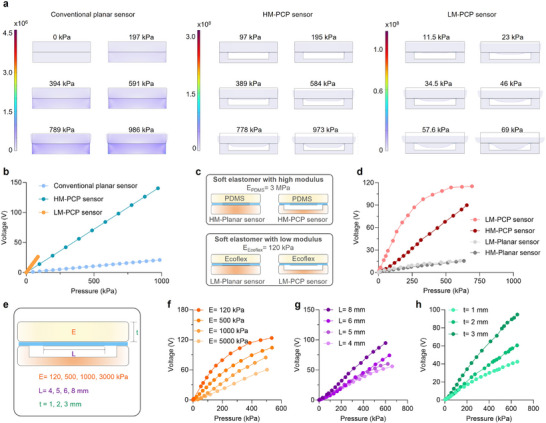
Structural optimization of the PCP sensor. a) Finite element simulation of stress distribution and b) corresponding simulated voltage as function of pressure for conventional planar piezoelectric sensor and PCP sensors using PDMS and Ecoflex as soft elastomers. c) Schematic diagrams of four representative sensors based on soft elastomers with various modulus, and d) corresponding experimental output voltage as function of external pressure. e) Schematic of structural parameters including elastomer modulus (*E*), electrode length (*L*), and elastomer thickness (*t*). f) Voltage‐pressure response of sensors based on elastomers with different elastic moduli. g) Voltage‐pressure response of sensors with different electrode length. h) Voltage‐pressure response of sensors with different elastomer thickness.

To further optimize the output performance of the PCP sensor, we further investigated the influence of soft elastomer modulus and geometrical parameters on output voltage. Finite element simulations were conducted on PCP sensors using two representative elastomers including PDMS (high modulus, 3 MPa) and Ecoflex (low modulus, 120 kPa) under various pressure, named as HM‐PCP and LM‐PCP sensors, respectively (Figure 2a). Compared to conventional planar sensor, the grooved architectures contribute to higher stress concentrations at the film edges, particularly in LM‐PCP sensor, indicating more effective strain transfer into the suspended piezoelectric layer. The conventional planar sensor exhibits minimal deformation even under high pressures. In contrast, the HM‐PCP sensor demonstrates enhanced stress transfer, while the LM‐PCP sensor shows pronounced film deformation and stress concentration under low pressures. These results suggest that the softer elastomers contribute to higher deformation at low loads, thereby enhancing sensitivity through mechanical amplification. Corresponding voltage‐pressure responses of the three sensors are simulated as shown in Figure 2b, demonstrating a monotonic and linear increase in output with pressure. LM‐PCP sensor exhibits higher output voltage under identical pressure condition due to the larger deformation in soft elastomer.

To further investigate the influence of structural configurations on sensing performance, four representative sensor types were designed by combining elastomers of different moduli with distinct geometries, including conventional planar sensor based on PDMS and Ecoflex (denoted as HM‐Planar and LM‐Planar sensor, respectively), and PCP sensor based on PDMS and Ecoflex (denoted as HM‐PCP and LM‐PCP sensor, respectively) (Figure 2c). As shown in Figure 2d, the PCP sensors generate significantly higher output voltages than planar sensors. Their response curves display a sharp initial rise followed by a plateau, whereas planar sensors exhibit slower and more linear voltage growth, due to limited strain. These trends are consistent with the simulation results shown in Figure 2b.

Figure 2e displays the key structural parameters that influence sensor performance, including the elastic modulus (*E)* of the soft elastomer, the electrode length (*L*), and the elastomer thickness (*t*). As shown in Figure 2f, the modulus of the soft elastomer plays a critical role in the stress transfer process. Low modulus elastomers exhibit premature saturation under high pressure, leading to reduced sensitivity and a narrower working range. In contrast, high‐modulus elastomer effectively delays strain saturation, consequently broadening the sensor's working range. By modulating the modulus of elastomer, a balance between sensitivity and detection range can be achieved.

The effect of electrode length (*L*) on sensor performance was investigated by fabricating sensors with active areas of 4 × 3 mm^2^, 5 × 3 mm^2^, 6 × 3 mm^2^, and 8 × 3 mm^2^ (Figure S4). As shown in Figure 2g, all four sensors exhibited good linearity and sensitivity across a broad pressure range. The sensor with *L* = 8 mm achieved a sensitivity of 167 mV·kPa^−1^ and excellent linearity (*R* ^2^ = 0.99 within 0–600 kPa). This enhancement can be attributed to the suppression of edge effects. Nonuniform electric‐field distribution near the electrode boundaries typically degrades signal linearity. Increased electrode area (e.g., from 4 × 3 mm^2^ to 8 × 3 mm^2^) mitigates the edge effect and improves the output voltage. The influence of elastomer thickness (t = 1, 2, and 3 mm) on HM‐PCP sensor performance is shown in Figure 2h. It indicates that thicker soft elastomers lead to higher output voltages under the same applied pressure due to the larger deformation, as verified by the simulated results in Figure S5. The impact of thickness on LM‐PCP sensors is provided in Figure S6, further validating this trend. These results highlight that the performance of sensor can be effectively tailored by engineering the modulus, geometry of the soft elastomer, and dimensional parameters of the grooved structure. The synergy between soft elastomer, grooved configurations, and optimized electrode designs offers a robust strategy for developing high‐sensitivity, wide‐range piezoelectric tactile sensors.

### 2.3 Dynamic Response of Optimized PCP Sensor

**FIGURE 3:**
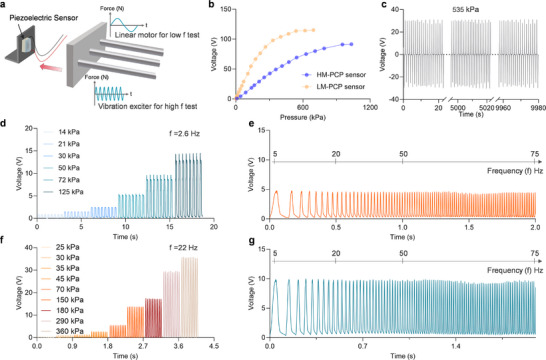
Dynamic response of optimized PCP sensor. a) Schematic of the experimental setup, where a linear motor was used for low‐frequency excitation and a vibration exciter for high‐frequency excitation. b) Sensing performance comparison of HM‐PCP and LM‐PCP sensors. c) Durability test of the HM‐PCP sensor under a periodic pressure of 535 kPa. d) Output voltage under varying pressure at low frequency (2.6 Hz). e) Frequency‐dependent output spectra at small dynamic pressures. f) Voltage responses under varying pressure at a high dynamic frequency (22 Hz). g) Frequency‐dependent output spectra at large dynamic pressures, illustrating the broadband dynamic sensing capability of the PCP sensor.

To evaluate the dynamic response characteristics and long‐term stability of the PCP tactile sensor under different working conditions, a dynamic loading platform was constructed based on a high‐precision linear motor system (Figure 3a). By integrating a frequency control module, cyclic pressure loading within a range of 5–75 Hz was achieved, enabling simulation of both low‐frequency quasi‐static stimuli and high‐frequency dynamic vibrations. In order to exclude possible triboelectric contributions, we performed a series of control experiments. The output voltage of the PCP sensor shows a highly linear dependence on applied pressure across contact and contact‐separation modes (Figure S7). The device based on depolarized non‐piezoelectric PVDF films further confirmed the extremely weak triboelectric signals and their negligible influence on the sensor output (Figure S8). In addition, reversing the poling direction led to a complete polarity reversal of the output voltage signal (Figure S9), confirming the piezoelectric response rather than triboelectric response.

To investigate the output performance of the sensors under high‐pressure dynamic loading, both HM‐PCP and LM‐PCP sensors were subjected to cyclic pressures ranging from 0 to 1000 kPa. As shown in Figure 3b, HM‐PCP sensor can maintain effective stress transfer under high‐pressure loads, thereby suppressing early voltage saturation and extending the operational pressure range. The HM‐PCP sensor achieves a broad working range up to 1 MPa with a sensitivity of 160 mV·kPa^−^^1^ in the 0–550 kPa range. The LM‐PCP sensor shows higher sensitivity of 330 mV·kPa^−^^1^ in the low‐pressure range (0–300 kPa). However, due to its tendency to undergo structural saturation at higher pressures, its sensitivity decreases to 80 mV·kPa^−^^1^ in the 350–500 kPa range. The sensor approaches complete saturation near 500 kPa, thus limiting its detection range. These results confirm a trade‐off between sensitivity and linear sensing range dominated by the modulus of the soft elastomer.

Long‐term stability is a critical criterion for the practical reliability of tactile sensors. Both the HM‐PCP and LM‐PCP sensors were connected to an external 50 MΩ load and subjected to 10 000 continuous loading cycles at 535 kPa. The results in Figure 3c demonstrate that the HM‐PCP sensor maintains a stable output voltage (∼30 V) throughout the test without noticeable signal degradation, indicating outstanding mechanical robustness and durability. It suggests that the sensor is resistant to mechanical fatigue under sustained high pressure. The corresponding cyclic test results for the LM‐PCP sensor are provided in Figure S10. The long‐term durability indicates its potential for applications in continuous monitoring scenarios.

The dynamic piezoelectric response behavior was further investigated by recording the voltage variation under pressures and frequencies. At low‐frequency excitation (2.6 Hz) with stepwise loading from 14 to 125 kPa, the HM‐PCP sensor exhibits step‐like voltage outputs (Figure 3d), indicating excellent pressure resolution capability. Frequency response was evaluated under constant pressure across 5–75 Hz (Figure 3e). The sensor generates stable voltage outputs with increasing frequency of excitation, confirming reliable frequency adaptability. Even at high‐frequency excitation (22 Hz) with large pressure amplitudes up to 360 kPa, the real‐time response to periodic pressure indicates excellent reproducibility of the sensor (Figure 3f). Under dynamic loading and unloading from 5 to 75 Hz, the output voltage remained stable at ∼10 V without degradation (Figure 3g). The dynamic piezoelectric response behavior of LM‐PCP sensor was also investigated as shown in Figures S11 and S12, indicating excellent cycling stability and durability. The PCP sensors exhibit superior performance in terms of sensitivity, detection range, dynamic response, and mechanical stability, validating its potential application in real‐time dynamic monitoring and wearable electronics.

### 2.4 Energy Harvesting Performance of the PCP Sensor

**FIGURE 4:**
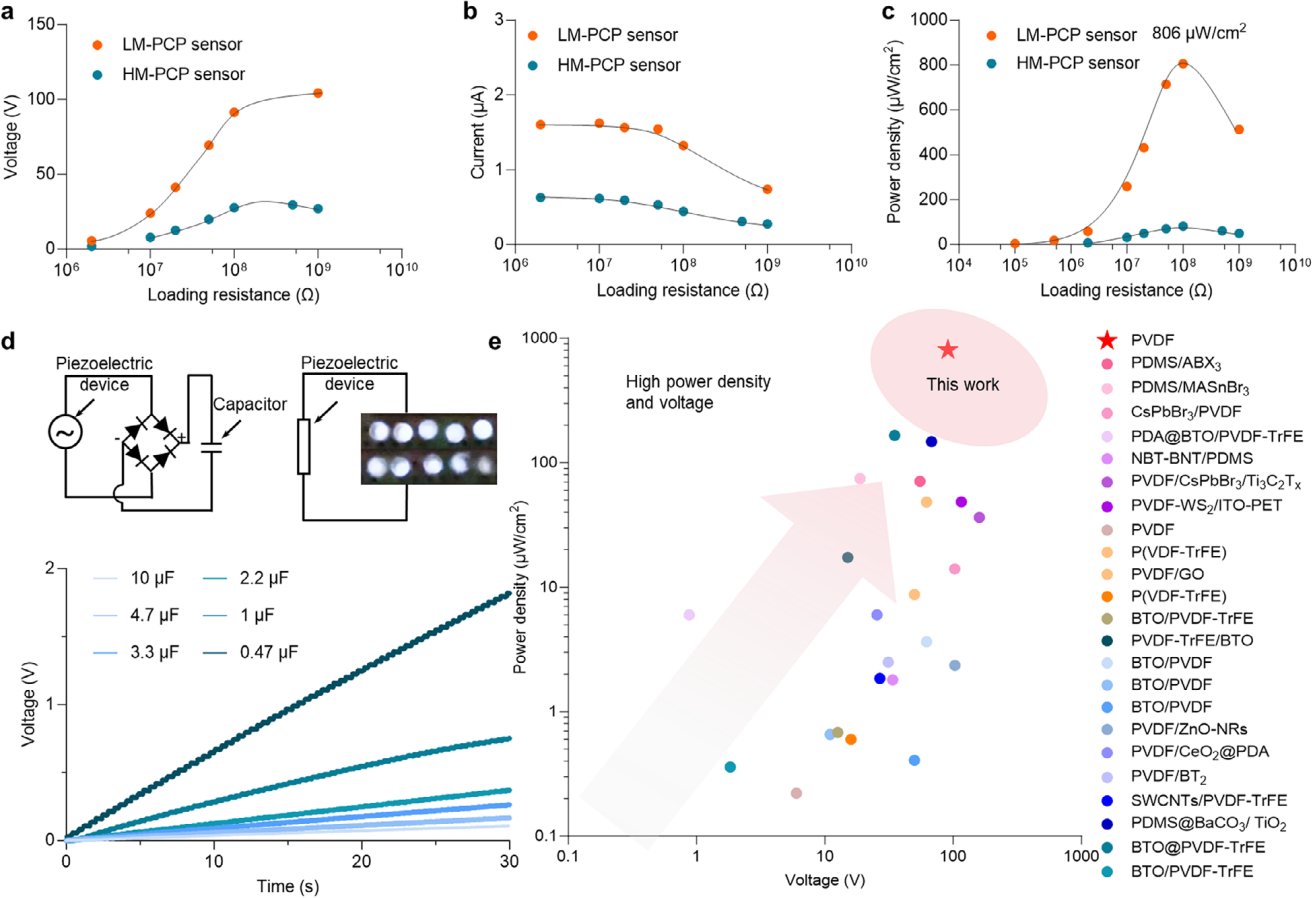
Energy harvesting performance of the PCP sensor. a) Output voltage, b) current and c) power of the LM‐PCP and HM‐PCP sensors as a function of external load resistances. d) LM‐PCP device can directly drive ten white LEDs under external mechanical stimulation and charge capacitors with capacitances ranging from 0.22 to 10 µF. e) Comparison of the maximum power density of our sensor with previously reported piezoelectric devices.

The power density of the piezoelectric devices highlights their tremendous potential for energy harvesting applications. To further evaluate the energy harvesting capability of the fabricated piezoelectric devices, the output performances of LM‐PCP and HM‐PCP sensors are systematically evaluated under varying external load resistances. The sensors were connected in series with external load resistors ranging from 0.1 MΩ to 1 GΩ and subjected to periodic mechanical stimulation. As shown in Figure 4a, the open‐circuit voltage of both PCP sensors increased with increasing load resistance. This is attributed to the fact that higher resistance limits current flow, facilitating charge accumulation at the electrode terminals, thereby enhancing the piezoelectric output. The LM‐PCP sensor exhibits higher output voltages under the same conditions, which is ascribed to the larger tensile strain of piezoelectric film caused by the low modulus‐indued greater deformation.

As shown in Figure 4b, the output current of the both sensors decreases gradually as the load resistance increases. Based on the measured voltage and current values, the output power at various load resistance can be calculated (Figure 4c). The LM‐PCP sensor achieved a maximum power output of 121 µW under a load resistance of 100 MΩ, corresponding to a power density of 806 µW·cm^−^^2^. The HM‐PCP sensor exhibits a peak power of 20 µW and a corresponding power density of approximately 133 µW·cm^−^^2^. The LM‐PCP sensor integrated with a rectifier circuit can be exploited as a self‐powering system for capacitor charging and lighting up a series of white LEDs. As shown in Figure 4d, the LM‐PCP sensor successfully powered 10 white LEDs under periodic mechanical excitation. Meanwhile, the sensor was connected to capacitors with different capacitance ranging from 0.22 to 10 µF for energy storage testing. As illustrated in Figure 4e, the sensor was able to charge a 0.47 µF capacitor to 1.5 V within 25 seconds under cyclic mechanical loadings. In addition, the output voltage and power density of the fabricated sensors was compared with representative piezoelectric devices reported in recent literature. As summarized in Figure 4e, our PCP sensor exhibits a super‐high voltage and power density, outperforming most state‐of‐the‐art piezoelectric devices [13, 14, 15, 16, 17, 18, 19, 20, 21, 22, 23, 24, 25, 26, 27, 28, 29, 30, 31, 32, 33, 34, 35]. The PCP sensor achieves record‐high voltage and power density, serving as a promising and reliable self‐powered energy source and sensing platform for wearable electronics.

### 2.5 Continuous Arterial Pulse Monitoring and Cardiovascular Assessment Using PCP Sensors

**FIGURE 5:**
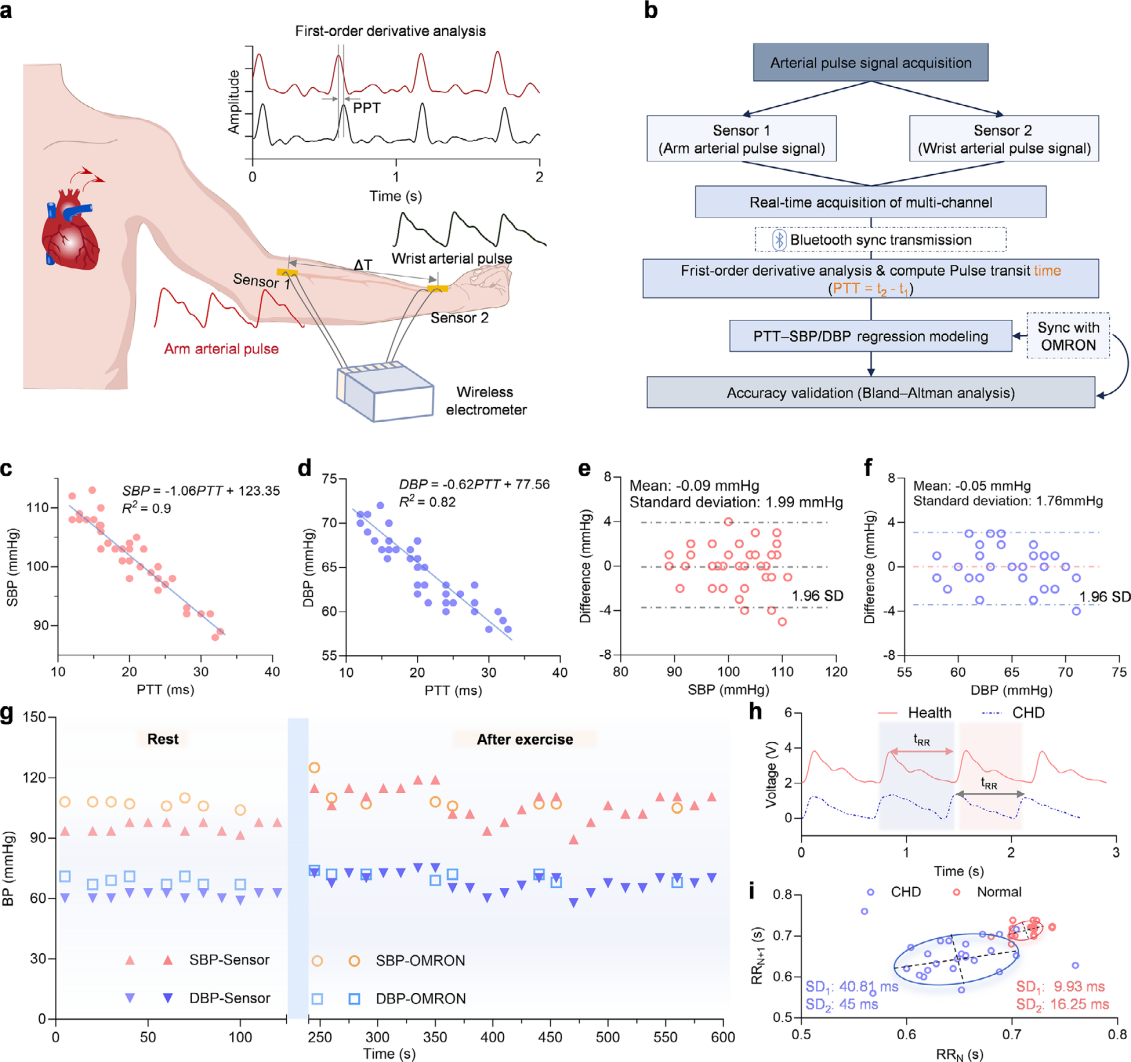
Continuous arterial pulse monitoring and cardiovascular assessment using PCP sensors. a) Schematic illustration of multi‐site pulse acquisition, where sensors adhered to the wrist and upper arm capture arterial pressure waves. The time delay between proximal (Sensor 1) and distal (Sensor 2) waveforms defines the pulse transit time (PTT). First‐order differentiation of the pulse signal highlights characteristic points for PTT calculation. b) Workflow of the integrated system, including multichannel acquisition, Bluetooth synchronization, first‐order differentiation for PTT extraction, regression modeling of systolic/diastolic blood pressure (SBP/DBP), and accuracy validation against a commercial sphygmomanometer. c, d) Correlation of PTT with SBP and DBP, derived from differentiated pulse waveforms. e, f) Bland‐Altman plots confirming the agreement of PCP sensor‐derived SBP and DBP with reference OMRON readings. g) Continuous blood pressure monitoring under rest and post‐exercise states, showing close consistency with OMRON values. h) Heart rate variability (HRV) analysis, where t_RR_ represents the interval between successive beats, enabling the identification of autonomic regulation. i) Poincaré plots comparing healthy subjects and coronary heart disease (CHD) patients, with SD_1_ and SD_2_ denoting short‐ and long‐axis standard deviations, revealing distinct cardiac dynamics.

To realize real‐time and continuous monitoring of vital signs, a blood pressure and pulse wave acquisition system based on PCP sensors was developed. Utilizing the ability of piezoelectric materials to generate electrical signals under slight mechanical pressure, the sensor enables continuous piezoelectric response acquisition of arterial pulse waves in real‐time. The PCP sensor can be attached to multiple anatomical sites including the auricular ridge, neck, arm, wrist, calf, and dorsum of the foot to capture the subtle skin deformations induced by pulsatile blood flow and vascular elasticity (Figure S13). Simultaneous measurements from the brachial artery (upper arm) and radial artery (wrist) enable extraction of pulse transit time (PTT), a hemodynamic parameter strongly correlated with blood pressure and vascular compliance (Figure 5a). To ensure portability and efficient data processing, the system integrates dual‐site sensors with a low‐power, Bluetooth‐enabled multi‐channel transmission module (Figure 5b), allowing synchronous multi‐site acquisition and wireless real‐time data processing for continuous cardiovascular assessment.

Pulse transit time (PTT), a time‐domain physiological parameter associated with vascular compliance, has been widely studied as a surrogate for noninvasive blood pressure estimation. When blood pressure increases, the arterial wall contracts, leading to fast blood flow and a short PTT. Conversely, when blood pressure decreases, the arterial wall relaxes, leading to slow blood flow and increased pulse transit time [36]. In this study, the time difference between characteristic points of two pulse waveforms was calculated to construct the relationship between PTT and blood pressure (BP) as follows:

(4) BP=aPTT+b

where a and b are coefficients. As shown in Figure 5c,d, both systolic (SBP) and diastolic blood pressure (DBP) exhibit strong negative correlations with PTT, confirming the intrinsic coupling between vascular stiffness and hemodynamic pressure.

To validate accuracy, Bland‐Altman analysis was performed by comparing PCP sensor‐derived values with a commercial OMRON sphygmomanometer (Figure 5e,f). The mean error was −0.09 ± 1.99 mmHg for SBP and 0.05 ± 1.76 mmHg for DBP, demonstrating close agreement between the two methods across five participants and further indicating that the sensor achieves accuracy and stability comparable to those of the OMRON device [37]. This error margin is well within clinical tolerance, underscoring the reliability of our flexible PCP sensor system for continuous blood pressure assessment. In addition to static validation, the dynamic blood pressure regulation before and after exercise was also examined (Figure 5g). At rest, the sensor recorded stable values of ∼96 mmHg SBP and ∼68 mmHg DBP. After exercising, the sensor successfully recorded an increase in systolic and diastolic pressure, reaching a peak of about 107 and 75 mmHg, respectively. The original pulse waveforms both at rest and after exercise can be seen in Figure S14. These results highlight the capability of the PCP sensor to track real‐time blood pressure fluctuations under varying physiological states, achieving noninvasive and continuous monitoring without the need for cuff‐based devices.

Beyond blood pressure, continuous pulse waveform analysis also enables assessment of heart rate variability (HRV), a well‐established marker of autonomic nervous system function and cardiovascular risk assessment [38]. From the extracted RR intervals, healthy individuals exhibit pronounced oscillatory pulse dynamics, whereas patients with coronary heart disease (CHD) show flatter waveforms with reduced fluctuation amplitudes (Figure 5h). Poincaré plots of RR interval sequences were constructed as shown in Figure 5i. Two key parameters, SD_1_ and SD_2_, are extracted to evaluate short‐term and long‐term heart rate variability, respectively. SD_1_ (standard deviation along the plot's short axis) and SD_2_ (along the long axis) reflect the degree of RR interval dispersion. Increases in these values indicate heightened variability and suggest instability in cardiac rhythm, serving as potential indicators of arrhythmia risk [39]. As shown in Figure 5i, RR_N_ values were plotted along the x‐axis and RR_N+1_ along the y‐axis to construct a 2D scatter. Healthy subjects exhibit compact distributions (SD_1_ = 9.93 ms, SD_2_ = 16.25 ms), while CHD patients show markedly increased dispersion (SD_1_ = 40.81 ms, SD_2_ = 45.00 ms), indicative of autonomic dysfunction and arrhythmogenic risk. Such quantitative HRV analysis provides a powerful tool for early identification of cardiovascular abnormalities.

### 2.6 High Fidelity Pulse Waveforms Acquisition for Cardiovascular Disease Evaluation

**FIGURE 6:**
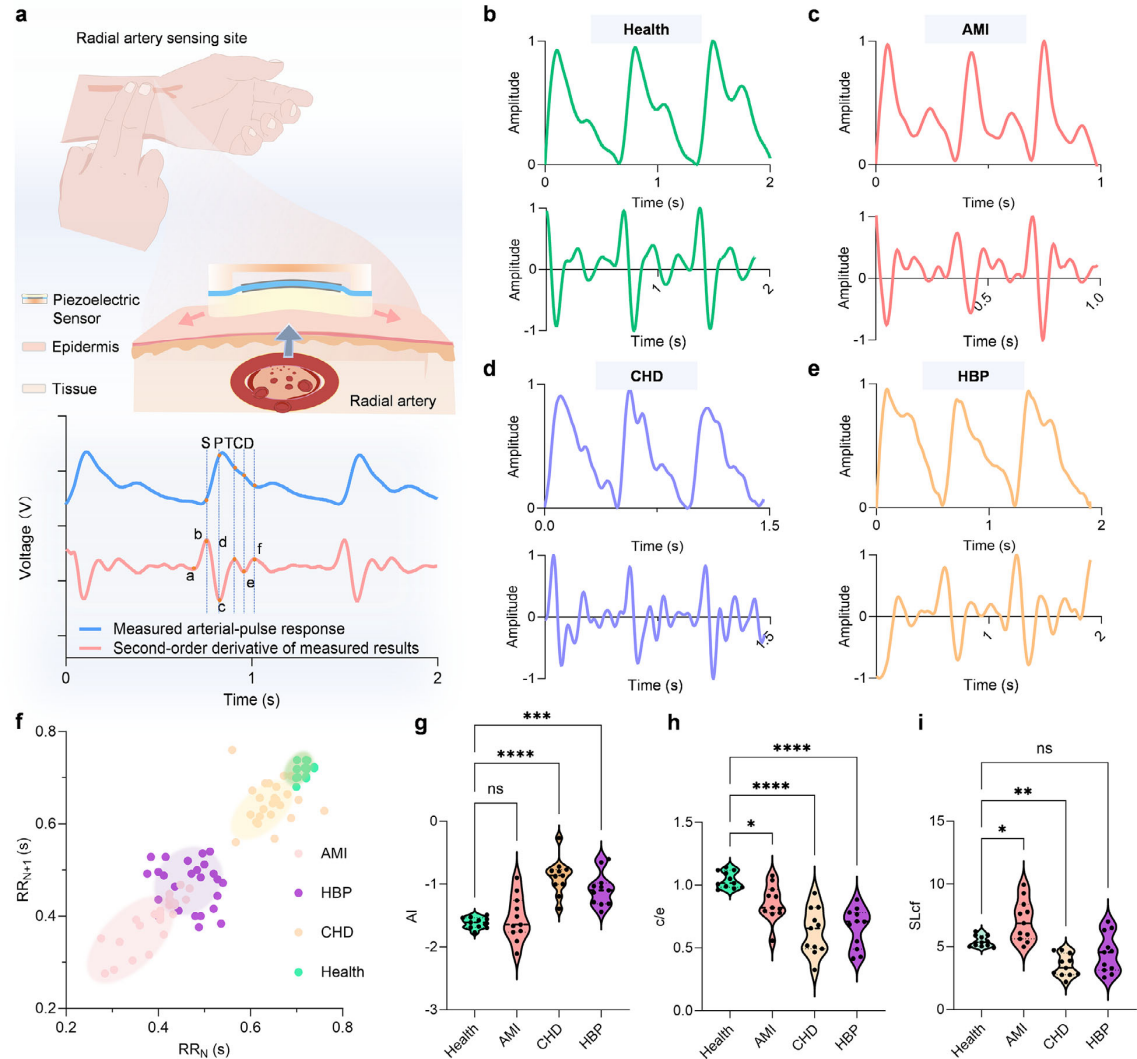
High fidelity pulse waveforms acquisition for cardiovascular disease evaluation. a) Schematic of radial artery monitoring using a PCP sensor. The measured arterial pulse waveforms (blue) and their second‐order derivatives (red) reveal key inflection points (S, P, T, C, D) and key features (a, b, c, d, e, and f) for hemodynamic interpretation. Representative pulse waveforms (top) and corresponding second‐order derivatives (bottom) for b) healthy subjects, c) patients with acute myocardial infarction (AMI), d) coronary heart disease (CHD), and e) hypertension (HBP). f) Heart rate variability (HRV) for four groups was assessed via Poincare plots using RR_N_ and RR_N+1_ intervals, highlighting intergroup distinctions. Hemodynamic indicators extracted from the pulse waveforms, including g) augmentation index (AI), h) left ventricular ejection capacity (c/e ratio), and i) arterial stiffness (SLcf). Statistical significance was evaluated by one‐way ANOVA with post hoc testing (*P < 0.05, **P < 0.01, ***P < 0.001, ****P < 0.0001; ns, not significant).

Arterial pulse waveform originates from rhythmic cardiac ejection and propagation through elastic arteries, carrying information related to vascular compliance, left ventricular function, and hemodynamics. High‐fidelity pulse waveforms recorded by the PCP sensor at the radial artery (Figure 6a) revealed distinct characteristic points (S, P, T, C, D) and derivative features (a‐f), which serve as critical landmarks for noninvasive functional assessment [39]. Significant differences in pulse waveforms are observed among different subjects. Figure 6b–e presents the pulse waveforms and corresponding second derivative signals from healthy subjects, acute myocardial infarction (AMI) patients, coronary heart disease (CHD) patients, and hypertension (HBP) patients. Healthy subjects show smooth, periodic waveforms, indicating good cardiovascular elasticity and hemodynamic stability. AMI patients exhibit sharp, densely packed waveforms with higher frequencies, reflecting myocardial ischemia‐induced cardiac dysfunction and disordered blood flow. In CHD patients, atherosclerosis increases the velocity of the tidal wave, leading to a reduced amplitude at point e, indicating reduced arterial elasticity and premature return of the reflected wave. HBP patients exhibit highly fluctuating waveforms, reflecting elevated vascular tension and strong hemodynamic impact.

Pulse‐derived heart rate variability (HRV) of the four groups was quantified using Poincaré plots (Figure 6f) [40]. Healthy individuals exhibit elliptical patterns consistent with balanced autonomic regulation, whereas AMI groups show greater dispersion due to the impaired cardiac autonomic control. CHD patients display relatively elliptical geometries, though subject variations may result in more dispersed data points. HBP patients exhibit scattered, irregular distributions, revealing long‐term impact of hypertension on the cardiovascular system and autonomic nervous regulation. The deviations in Poincare plot geometries can provide important indicators for the diagnosis and monitoring of cardiovascular diseases [41].

The distinctions from the four groups can be further verified by quantitative analysis. The Augmentation Index (AI), a key parameter for assessing arterial stiffness, was significantly elevated in CHD and HBP patients compared with healthy controls (P < 0.001, Figure 6g), reflecting early return of reflected waves and increased left ventricular afterload. The c/e ratio, an index of left ventricular ejection capacity, was significantly reduced in CHD and AMI patients (Figure 6h), indicating their impaired contractile function. In addition, the SLcf index related to cardiovascular function, HRV, or autonomic nervous system activity is useful for assessing arterial stiffness and cardiovascular disease states. The c point corresponds to the percussion wave resulting from left ventricular ejection transmitted through elastic arteries, while point f is the dicrotic wave caused by blood reentering the aortic valve under pressure, generating a reflective oscillatory wave. As shown in Figure 6i, the SLcf value in healthy subjects differs significantly from that of CHD patients with statistical significance (P < 0.01). These analyses confirm the capability of PCP‐sensor‐derived pulse waveforms to provide a comprehensive, noninvasive assessment of cardiovascular health. Various parameters reflect the dynamic changes in physiological states under both healthy and pathological conditions, demonstrating the diagnostic potential of pulse waveform parameters in the noninvasive early screening of cardiovascular diseases.

## 3 Conclusion

Inspired by the strain transduction principle of Pacinian corpuscles, we developed a piezoelectric tactile sensor that transforms vertical loading into lateral tensile strain through a grooved architecture, thereby amplifying local deformation and boosting electromechanical conversion. Finite element simulations and experimental results confirmed the advantages of the grooved structure in stress concentration and strain amplification that endow the PCP sensor with record‐high sensitivity, wide linear response, and robust durability. By engineering the modulus of encapsulating elastomers and optimizing electrode geometry, the device balances sensitivity and linear sensing range, overcoming long‐standing trade‐offs in tactile sensing. The sensor demonstrates practical utility in real‐time, noninvasive cardiovascular monitoring. Multi‐site pulse acquisition, cuffless blood pressure estimation, and analyses in heart rate variability and Poincaré mapping establish a multidimensional platform for assessing vascular compliance, autonomic regulation, and cardiac performance. This bioinspired strategy establishes a novel design principle for high‐performance piezoelectric sensors while unlocking new opportunities in wearable diagnostics and intelligent healthcare.

## 4 Experimental Section

### 4.1 Materials and Reagents

Polydimethylsiloxane (PDMS, Dow Corning Sylgard 184) was purchased from Dow Corning Corporation (USA). Ecoflex silicone rubber (produced by Smooth‐On) was supplied by Shenzhen Jinwang Technology Co., Ltd. Silver fiber conductive threads were obtained from Zhuji Jinshengkang Textile Technology Co., Ltd. Uniaxially stretched piezoelectric polyvinylidene fluoride (PVDF) films (70 µm thickness) were procured from Shenzhen Zhimei Technology Co., Ltd. Polymethyl methacrylate (PMMA) was also employed in this study.

### 4.2 Preparation of Soft Elastomer Encapsulation Layer

The precursor and curing agent of liquid PDMS were mixed at a weight ratio of 10:1, degassed under vacuum, and then poured into a mold prepared by laser engraving. After curing at 80°C in an oven for 30 min, the PDMS material was demolded and cut into pieces with dimensions of 17 × 8 mm^2^. For Ecoflex elastomer, Part A and Part B of Ecoflex‐0030 were mixed at a mass ratio of 1:1, poured into a laser‐engraved mold, and allowed to cure at room temperature for 20 min. The cured Ecoflex was then demolded and cut into pieces measuring 17 × 8 mm^2^. The prepared PDMS and Ecoflex materials were used separately as the soft encapsulation layer.

### 4.3 Preparation of PVDF‐Based Piezoelectric Electrodes

Conductive silver paste was uniformly brushed onto both sides of the piezoelectric PVDF film with a thickness of 28 µm to form silver electrode layers on the top and bottom surfaces. Each electrode layer had an area of X × 3 mm^2^, where the width was fixed at 3 mm, and the length X was set to 4, 5, 6, or 8 mm, respectively. Silver conductive wires were then stably adhered to one end of each silver electrode layer on both sides of the PVDF film using silver paste, serving as the second piezoelectric layer.

### 4.4 Preparation of the Rigid Grooved Frame and Assembly of the PCP Sensors

A PMMA plate with a groove was fabricated using a laser engraving machine. The outer frame had an area of 17 × 8 mm^2^, and the groove measured 12 × 5 mm^2^ with a depth of 2 mm, leaving a bottom thickness of 1 mm. To facilitate the placement of conductive wires, two small slots, each with an area of 1.3 × 1.5 mm^2^ and a depth of 0.5 mm, were additionally engraved at both ends of one long side of the groove. The prepared PMMA plate served as the third supporting layer. The three prepared layers were sequentially stacked and assembled to obtain piezoelectric pressure sensors encapsulated with either PDMS or Ecoflex, denoted as HM‐PCP and LM‐PCP sensor, respectively.

### 4.5 High Fidelity Pulse Waveforms Acquisition for Cardiovascular Disease Evaluation

For the sensor dedicated to pulse signal monitoring, the overall dimensions are set to 17 × 8 mm^2^, with an active electrode area of 5 × 3 mm^2^. The groove structure of the rigid frame is 12 × 5 mm^2^ (with a depth of 2 mm). To enable more accurate pulse data collection with skin‐conformal contact, a 1 mm‐thick PDMS elastomer is used for encapsulation. These parameters are standardized to ensure the reproducibility of fabrication and the stability of monitoring performance. To verify the practical applicability of the fabricated PCP sensors for physiological pulse signal monitoring, tests were conducted on healthy volunteers with informed consent obtained prior to the experiment.

### 4.6 Characterization and Measurements

Dynamic pressure was applied to the sensors using a LinMot linear motor performing periodic mechanical impact motions, with the force magnitude recorded in real time by a pressure gauge. During the pressure variation process, electrical signals, including voltage, current, and charge, were measured using an electrometer (Keithley 6514). To assess the dynamic response and mechanical stability, cyclic loading tests were conducted under a pressure of 535 kPa for up to 10 000 cycles. Frequency‐dependent performance was evaluated under operating frequencies ranging from 2.6 to 75 Hz. Finite element analysis (FEA) was performed on COMSOL Multiphysics 6.2 to simulate the stress distribution and strain behavior within the PVDF layer under various loading conditions.

## Author Contributions

Zhongqian Song and Li Niu proposed and supervised the project. Qi Yang, Zhongqian Song, and Yu Bao conceived and designed the experiments. Qi Yang, Zhongqian Song, Shengjie Liu, Yingming Ma, and Weiyan Li performed the experiments and analyzed the data. Zhongqian Song, Huijun Kong, Cuiyu Liu, and Yu Bao gave guidance on the data analysis and paper revision. Qi Yang, Zhongqian Song, Yu Bao, and Li Niu wrote the manuscript.

## Conflicts of Interest

The authors declare no conflicts of interest.

## Supporting information




**Supporting File**: advs74224‐sup‐0001‐SuppMat.docx.

## Data Availability

The data that support the findings of this study are available from the corresponding author upon reasonable request.
