# Supplementary material for: Pacinian Corpuscle‐Inspired Strain Conversion Enables Ultrasensitive, Linear, and Broad‐Range Piezoelectric Sensing for Cardiovascular Health Monitoring
Source: Adv Sci (Weinh). 2026 Feb 3;13(21):e22157. doi: 10.1002/advs.202522157 (PMC13073301; doi:10.1002/advs.202522157)
Supplement: Supplementary file 1 — Supporting File: advs74224‐sup‐0001‐SuppMat.docx. [file ADVS-13-e22157-s001.docx]

**Supplementary information**

**Pacinian Corpuscles-Inspired Tactile Sensor with Enhanced Piezoelectric Response for Cardiovascular Assessment**

*Qi Yang^a^, Zhongqian Song^b,^* , Shengjie Liu^c^, Yingming Ma^a^, Weiyan Li^b^, Huijun Kong^b^, Cuiyu, Liu^b^, Yu Bao^a,^*, Li Niu^c,^**

a Center for Advanced Analytical Science, c/o School of Chemistry and Chemical Engineering, Guangzhou University, Guangzhou 510006, P.R. China

b College of Medical Information and Artificial Intelligence, Shandong First Medical University & Shandong Academy of Medical Sciences, 250117, P.R. China

c School of Chemical Engineering and Technology, Sun Yat-sen University, Zhuhai 519082, P.R. China


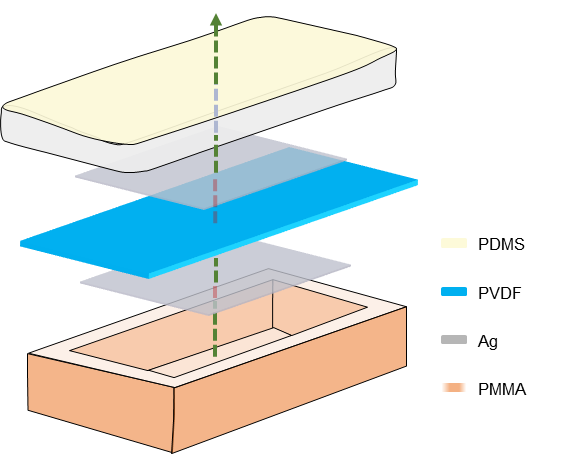


Figure S1. The schematic diagram of the sensor’s layered structure

The sensor consists of three primary components: a soft elastomer, conductive electrode layers, and a rigid supporting framework. The bottom supporting layer consists of a rigid framework with a rectangular groove, over which the piezoelectric sensing unit composed of a polyvinylidene fluoride (PVDF), bridging both edges of the groove.

When external pressure is applied to the piezoelectric sensor, the piezoelectric material undergoes mechanical deformation, leading to internal polarization and the generation of an output voltage. The relationship between the applied pressure and the resulting voltage is derived as follows.

| $F_{t} = \sigma_{31}\cdot d_{31}\cdot w$ | (1) |
| --- | --- |

According to the constitutive relation of piezoelectric materials, the stress component can be expressed as the product of the elastic modulus *E* and the mechanical strain$\varepsilon$, as defined by Hooke’s law.

| $\sigma_{31}= E\cdot\varepsilon$ | (2) |
| --- | --- |

where E represents Young’s modulus of material.

Based on the above relation, the tensile force F_t_ can be obtained as follows.

| $F_{t} = E\cdot\varepsilon\cdot d_{31}\cdot w$ | (3) |
| --- | --- |

The generation of electrical charge in a piezoelectric material is governed by the direct piezoelectric effect, in which mechanical stress induces electric polarization. The relationship between the generated charge Q and the applied stress can be described by the following expression.

| $Q = d_{31}\cdot\sigma_{31}\cdot S$  $= d_{31}\cdot\sigma_{31}\cdot l\cdot w$  $= d_{31}E\cdot\varepsilon\cdot l\cdot w$ | (4) |
| --- | --- |

The capacitance C of piezoelectric materials is typically calculated based on the parallel-plate capacitor model, which is applicable to most membrane-type piezoelectric sensor structures.

| $C = \frac{\varepsilon_{0}\cdot\varepsilon_{r}\cdot S}{d}$ | (5) |
| --- | --- |

The expression for output voltage can be derived.

| $V = \frac{Q}{C}$  $= \frac{d_{31}\cdot\sigma_{31}\cdot S}{\frac{\varepsilon_{0}\cdot\varepsilon_{r}\cdot S}{d}}$  $= \frac{d_{31}\cdot\sigma_{31}\cdot d}{\varepsilon_{0}\cdot\varepsilon_{r}}$ | (6) |
| --- | --- |

Therefore, the output voltage depends on the piezoelectric constant e and the elastic modulus E. When the elastic modulus is higher and the strain is larger, the output voltage will also increase.

The representative summary is as follows.

| **Symbol** | **Description** | **Unit** |
| --- | --- | --- |
| *σ*_31_​ | Piezoelectric coefficient (charge generated in direction 1 per stress in direction 3) | Pa |
| d | Thickness of the piezoelectric material | m |
| w | Effective width of the piezoelectric material | m |
| E | Young's modulus | Pa |
| Q | Generated electric charge | C |
| $\text{d}_{\text{31}}$ | Piezoelectric coefficient (charge per unit force or strain per unit voltage) | C/N or m/V |
| $\text{ε}$ | Strain in the materia | Dimensionless |
| $\text{S}$ | Electrode area | m² |
| $\text{ε}$*_0_* | Vacuum Permittivity | F/m |
| $\text{ε}$_r_ | Relative Permittivity | Dimensionless |

**
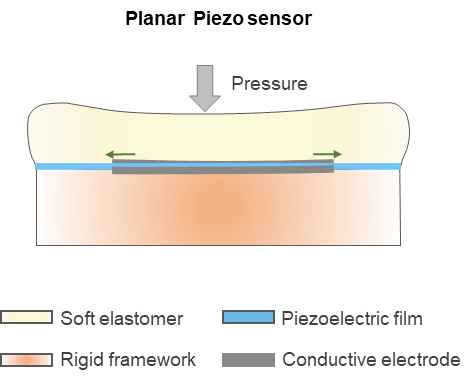
**

Figure S2. The schematic diagram of the planar sensor.

**
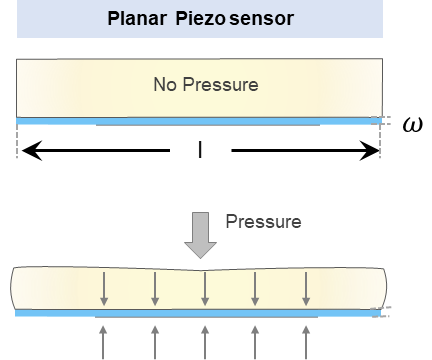
**

Figure S3. Deformation and working mechanism of the planar-structure piezoelectric sensor under pressure.

**
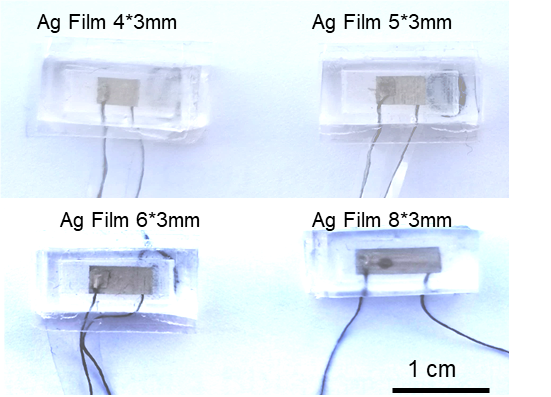
**

Figure S4. Sensors with active areas of 4×3 mm², 5×3 mm², 6×3 mm², and 8×3 mm².

**
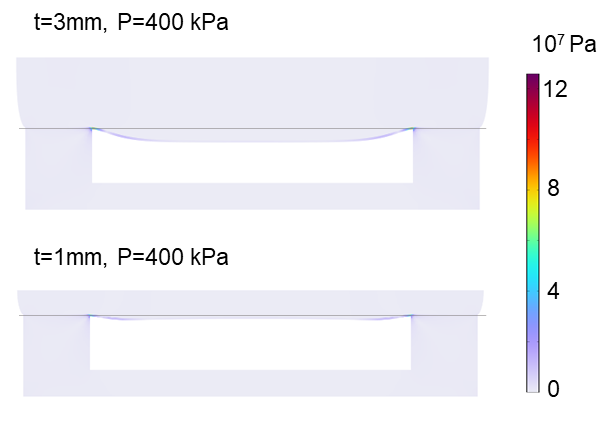
**

Figure S5. Finite element simulations of PCP sensor with different elastomer thickness under 400 kPa loading.

Figure S5 show that, under the same pressure of 400 kPa, a thicker elastomer (3 mm) exhibits greater vertical and lateral deformation in piezoelectric film than a thinner one (1 mm). This occurs because a thicker soft layer has a larger effective compliance and strain accommodation volume, enabling larger deformation and lateral strain in the piezoelectric film. The increased lateral stretch enhances dipole alignment and charge generation, thereby producing a higher output voltage.

Figure S6. Pressure response curves of HM-PCP sensor under encapsulation thickness.


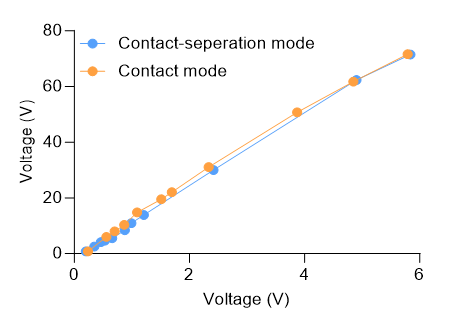


Figure S7. Output voltage of PCP sensor under contact and contact-separation testing modes.

The output performance of the PCP sensor obtained by contact and non-contact testing is consistent. Thus, the triboelectric influence caused by contact during the testing process can be neglected.


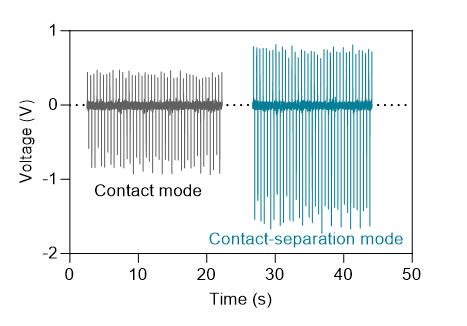


Figure S8. Output voltage of non-piezoelectric PVDF films under contact and contact-separation testing mode.


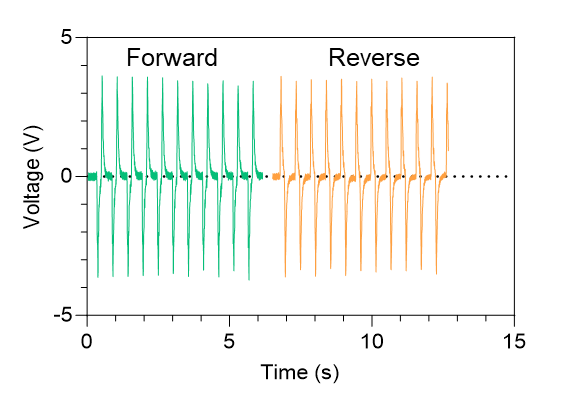


Figure S9. Comparison of output voltage signals from PCP sensors with opposite PVDF polarization directions, indicating that the observed high output voltage originates from the piezoelectric effect rather than triboelectric mechanisms.

**
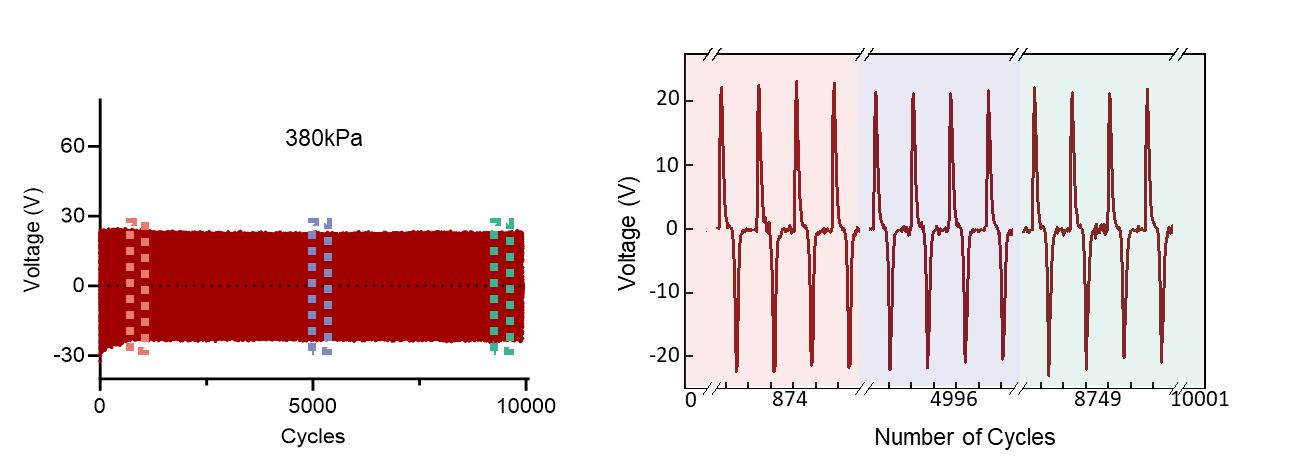
**

Figure S10. The stability test results of the HM-PCP sensor, the left image is a magnified view of a local area.

Figure S11. Piezoelectric response of the HM-PCP sensor under varying applied pressures.

Figure S12. Output voltage response of the LM-PCP sensor under different frequencies.

**
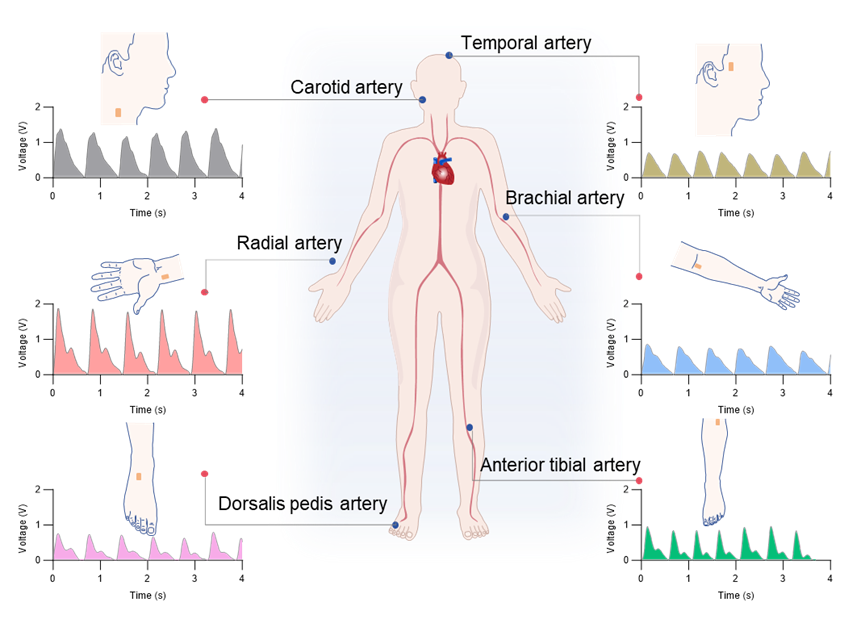
**

Figure S13. Schematic illustration of arterial pulse wave measurement in the human body.


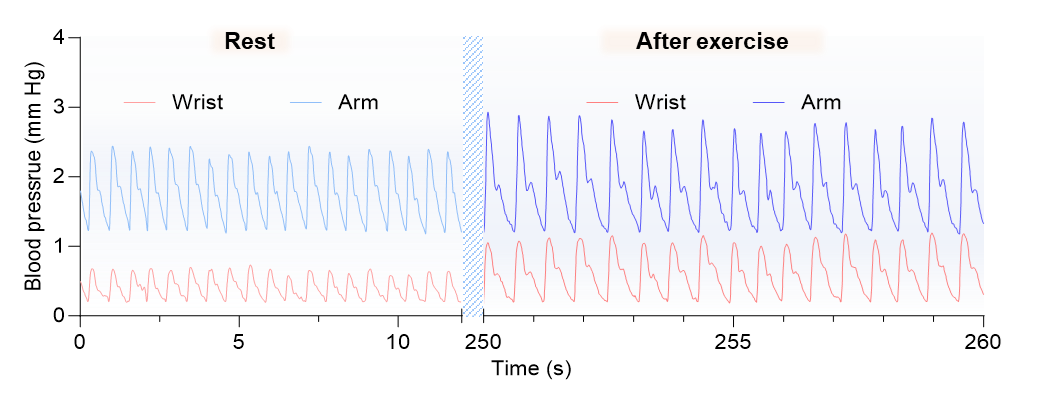


Figure S14. The original pulse waveforms both at rest and after exercise.

The arterial pulse waveform consists of several characteristic points, each reflecting specific aspects of cardiac ejection and arterial elasticity. The onset of the waveform, marked by point S (b), corresponds to the rapid ejection of blood into the aorta during systole. The percussion wave (point c, P wave) represents the primary peak caused by the sudden rise in arterial pressure and is closely associated with left ventricular ejection function and arterial compliance, with a higher peak amplitude indicating stronger cardiac pumping. The tidal wave (point e, C wave) occurs during the late phase of ventricular ejection, while the trough (point d, T wave) reflects the nadir of the reflected wave. Finally, the dicrotic wave (point f, D wave) is associated with the reopening of the atrioventricular valves and rebound of blood flow.

Based on these characteristic points, several hemodynamic indices can be derived. The Acceleration Index (AI) is defined as

| $\text{AI=}\frac{\text{Ac-Ad-Ae-}\text{Af}}{\text{Ab}}$ | (1) |
| --- | --- |

where Ai refers to the amplitude of characteristic points on the pulse waveform, AI provides a measure of the acceleration of left ventricular ejection.

The c/e value serves as an indicator of ventricular ejection performance, where a higher value suggests stronger left ventricular ejection function, defined as

| $\text{c/e=Ae-Ac}$ | (2) |
| --- | --- |

To further evaluate late systolic function, the Systolic Late Function index (SLcf) is introduced

| $\text{SLcf}\text{=}\frac{\text{Af}\text{-Ac}}{\text{Tf}\text{-Tc}}$ | (3) |
| --- | --- |

where A and T represent the amplitude and timing of characteristic points c and f, respectively. This index reflects the hemodynamic properties associated with the tidal wave and dicrotic wave, thereby providing insight into late systolic ventricular dynamics.

Table S1. Summary of sensor performance reported in recent literature.

| **Piezoelectric materials** | **Voltage**  **(V)** | **Power density**  **(μW/cm^2^)** | **Detection Range**  **(kPa)** | **Sensitivity**  **(mV/kPa)** | **Ref.** |
| --- | --- | --- | --- | --- | --- |
| PVDF/CeO_2_@PDA | 25.5 | 6 | 10-50  50-100 | 85  20 | ^[1]^ |
| NBT-BNT/PDMS | 33.8 | 1.8 | 0-52 | 1865 | ^[2]^ |
| PVDF/BT_2_ | 31.2 | 2.5 | 0-7.5  7.5-25 | 2088  1092 | ^[3]^ |
| PVDF-TrFE/BTO | 15.1 | 17.33 | - | - | ^[4]^ |
| P(VDF-TrFE) | 50 | 8.75 | - | - | ^[5]^ |
| PDA@BaTiO_3_/P(VDF-TrFE) | 6 | 0.878 | - | - | ^[6]^ |
| P(VDF-TrFE) | 16 | 0.6 | 25-75 | ~167.12 | ^[7]^ |
| PVDF | 6 | 0.221 | 133 | 103.5 | ^[8]^ |
| BaTiO_3_@PVDF-TrFE | 59.5 | 1.658 | 111 | - | ^[9]^ |
| PVDF-WS_2_/ITO-PET | 116 | 48.5 | 3.1-105 |  | ^[10]^ |
| PVDF/graphene oxide | 62 | 48.3 | - | - | ^[11]^ |
| PVDF/CsPbBr_3_/Ti_3_C_2_T_x_ | 160 | 36.4 | - | - | ^[12]^ |
| PDMS/ABX_3_ | 55.2 | 70.9 | - | - | ^[13]^ |
| PDMS  @(BaCO_3_/TiO_2_) | 68 | 148 | 2.5-100 | 472 | ^[14]^ |
| BTO/PVDF | 62 | 3.64 | - | - | ^[15]^ |
| SWCNTs/PVDF-TrFE | 26.8 | 1.85 | - |  | ^[16]^ |
| BTO/PVDF-TrFE | 1.83 | 0.36 | - | 33 | ^[17]^ |
| BTO/PVDF | 11 | 0.656 | - | - | ^[18]^ |
| CsPbBr_3_/PVDF | 103 | 14 | - | - | ^[19]^ |
| BTO/PVDF | 50 | 0.407 | - | - | ^[20]^ |
| PVDF/ZnO | 103.1 | ∼2.36 | 22 | - | ^[21]^ |
| PDMS/MASnBr_3_ | ∼18.8 | ∼74.52 | 0-500 | - | ^[22]^ |
| BTO/PVDF | 12.6 | 0.7 | - | - | ^[23]^ |
| PVDF | 91 | 806 | 0-600 | 185 | Our work |

Reference

[1] S. Huang, X. Zhao, C. Hao, Z. Ma, H. Wang, A. Zhang, W. Zhang, L. Li, W. Zhang, *Chemical Engineering Journal* **2024**, *482*, 148950.

[2] H. Yin, Y. Guan, Y. Li, Z. Zheng, Y. Guo, *Nano Energy* **2023**, *118*, 109044.

[3] Z. Shao, X. Zhang, J. Liu, X. Liu, C. Zhang, *Small Methods* **2023**, *7*, 2300701.

[4] R. Peng, B. Zhang, G. Dong, Y. Wang, G. Yang, J. Zhang, B. Peng, Y. Zhao, M. Liu, *Advanced Functional Materials* **2024**, *34*, 2316519.

[5] L. Zhang, J. Gui, Z. Wu, R. Li, Y. Wang, Z. Gong, X. Zhao, C. Sun, S. Guo, *Nano Energy* **2019**, *65*, 103924.

[6] X. Guan, B. Xu, J. Gong, *Nano Energy* **2020**, *70*, 104516.

[7] F. Lv, J. Lin, Z. Zhou, Z. Hong, Y. Wu, Z. Ren, Q. Zhang, S. Dong, J. Luo, J. Shi, R. Chen, B. Liu, Y. Su, Y. Huang, *Nano Energy* **2022**, *100*, 107507.

[8] L. He, J. Lu, C. Han, X. Liu, J. Liu, C. Zhang, *Small* **2022**, *18*, 2200114.

[9] Y. Cho, J. Jeong, M. Choi, G. Baek, S. Park, H. Choi, S. Ahn, S. Cha, T. Kim, D.-S. Kang, J. Bae, J.-J. Park, *Chemical Engineering Journal* **2022**, *427*, 131030.

[10] D. Bhattacharya, S. Bayan, R. K. Mitra, S. K. Ray, *Nanoscale* **2021**, *13*, 15819-15829.

[11] S. Tiwari, A. Gaur, C. Kumar, P. Maiti, *Sustainable Energy & Fuels* **2020**, *4*, 2469-2479.

[12] Y. Xue, T. Yang, Y. Zheng, K. Wang, E. Wang, H. Wang, L. Zhu, Z. Du, H. Wang, K.-C. Chou, X. Hou, *Advanced Science* **2023**, *10*, 2300650.

[13] S. Deswal, S. K. Singh, R. Pandey, P. Nasa, D. Kabra, B. Praveenkumar, S. Ogale, R. Boomishankar, *Chemistry of Materials* **2020**, *32*, 8333-8341.

[14] X. Wei, K. Xu, Y. Wang, Z. Zhang, Z. Chen, *ACS Applied Materials & Interfaces* **2024**, *16*, 11740-11748.

[15] Y. K. Kim, S.-H. Hwang, H.-J. Seo, S. M. Jeong, S. K. Lim, *Nano Energy* **2022**, *97*, 107216.

[16] K. Kim, S. Lee, J.-S. Nam, M. Joo, B. Mikladal, Q. Zhang, E. I. Kauppinen, I. Jeon, S. An, *Advanced Functional Materials* **2023**, *33*, 2213374.

[17] X. Zhou, K. Parida, J. Chen, J. Xiong, Z. Zhou, F. Jiang, Y. Xin, S. Magdassi, P. S. Lee, *Advanced Energy Materials* **2023**, *13*, 2301159.

[18] K. Shi, B. Sun, X. Huang, P. Jiang, *Nano Energy* **2018**, *52*, 153-162.

[19] H. Chen, L. Zhou, Z. Fang, S. Wang, T. Yang, L. Zhu, X. Hou, H. Wang, Z. L. Wang, *Advanced Functional Materials* **2021**, *31*, 2011073.

[20] B. S. Athira, A. George, K. Vaishna Priya, U. S. Hareesh, E. B. Gowd, K. P. Surendran, A. Chandran, *ACS Applied Materials & Interfaces* **2022**, *14*, 44239-44250.

[21] Z. Wu, J. Huang, Y. Zhao, X. Ding, J. Chen, Z. Liu, Z. Liu, Y. Zhu, *Chemical Engineering Journal* **2025**, *504*, 158874.

[22] S. Ippili, V. Jella, J. Kim, S. Hong, S.-G. Yoon, *ACS Applied Materials & Interfaces* **2020**, *12*, 16469-16480.

[23] K. Shi, B. Chai, H. Zou, P. Shen, B. Sun, P. Jiang, Z. Shi, X. Huang, *Nano Energy* **2021**, *80*, 105515.
